# Gut microbiota regulate tumor metastasis via circRNA/miRNA networks

**DOI:** 10.1080/19490976.2020.1788891

**Published:** 2020-07-18

**Authors:** Zhuxian Zhu, Jianguo Huang, Xu Li, Jun Xing, Qiang Chen, Ruilin Liu, Feng Hua, Zhongmin Qiu, Yuanlin Song, Chunxue Bai, Yin-Yuan Mo, Ziqiang Zhang

**Affiliations:** a Department of Nephrology, Tongji Hospital, Tongji University School of Medicine, Shanghai, China; b Department of Radiation Oncology, Duke University Medical Center, Durham, NC, USA; c Department of General Medicine, Tongji University School of Medicine, Shanghai, China; d Department of Respiratory and Critical Care Medicine, and Department of Infectious Disease, Tongji Hospital, Tongji University School of Medicine, Shanghai, China; e Department of Respiratory Medicine, Affiliated Huzhou Hospital of Zhejiang University, Zhejiang, China; f Department of Respiratory and Critical Care Medicine, Zhongshan Hospital, Fudan University, Shanghai, China; g Cancer Institute and Department of Pharmacology/Toxicology, University of Mississippi Medical Center, Jackson, MS, USA

**Keywords:** Gut microbiota, cancer, metastasis, circular RNA (circRNA), microRNA, interleukin-11(IL-11), cancer stem cell

## Abstract

**Background:**

Increasing evidence indicates that gut microbiota plays an important role in cancer progression. However, the underlying mechanism remains largely unknown. Here, we report that broad-spectrum antibiotics (ABX) treatment leads to enhanced metastasis by the alteration of gut microbiome composition.

**Methods:**

Cancer LLC and B16-F10 cell metastasis mouse models, and microarray/RNA sequencing analysis were used to reveal the regulatory functions of microbiota-mediated circular RNA (circRNA)/microRNA (miRNA) networks that may contribute to cancer metastasis.

**Results:**

The specific pathogen-free (SPF) mice with ABX treatment demonstrated enhanced lung metastasis. Fecal microbiota transplantation (FMT) from SPF mice or Bifidobacterium into germ-free mice significantly suppressed lung metastasis. Mechanistically, gut microbiota impacts circRNA expression to regulate levels of corresponding miRNAs. Specifically, such modulations of gut microbiota inhibit mmu_circ_0000730 expression in an IL-11-dependent manner. Bioinformatics analysis combined with luciferase reporter assays revealed reciprocal repression between mmu_circ_0000730 and mmu-miR-466i-3p. We further showed that both mmu-miR-466i-3p and mmu-miR-466 f-3p suppresses a number of genes involved in epithelial-mesenchymal transition (EMT) and stemness of cancer stem cells such as SOX9.

**Conclusions:**

These results provide evidence of a previously unrecognized regulatory role of non-coding RNAs in microbiota-mediated cancer metastasis, and thus, the microbiome may serve as a therapeutic target.

## Introduction

Gut microbiota has been implicated in cancer.^1,2^ Evidence is growing that the gut microbiota modulates the host response to cancer therapeutics, such as primary resistance to chemotherapy or immunotherapy.^3–7^ Moreover, gut microbiota dysbiosis due to broad-spectrum antibiotics (ABX) during anticancer treatments may disturb the cancer microenvironment contributing to cancer progression.^8–10^ However, little is known about whether gut microbiota regulates tumor metastasis.

Metastasis is a complex process that requires the interaction between tumor cells and their microenvironment.^11^ Disturbance of gut microbiota composition is correlated with impaired immune cell activity,^12^ while microorganisms such as probiotics can remodel the tumor microenvironment. However, little is known as to whether gut microbiota alters the tumor microenvironment by affecting circulating non-coding RNAs such as circular RNAs (circRNAs)/microRNAs (miRNAs) that contribute to cancer metastasis; and the underlying mechanism remains to be determined yet.

The gut microbiota affects inflammation and immunity not only locally at the mucosal level but also systemically,^13–15^ raising a question of whether the microbiota alters the tumor microenvironment by regulating circulating non-coding RNAs, including circRNAs and miRNAs, that may contribute to cancer metastasis and therapy efficacy. circRNAs are a novel class of endogenous non-coding RNAs (ncRNAs) formed from exons or introns through special selective shearing. Accumulating evidence indicates that circRNAs play a vital role in modulating tumor development by maintaining cellular homeostasis.^16^ The involvement of circRNAs in different types of cancers has also been reported.^17^ They can play a role of regulatory interaction with miRNA to prevent mRNA translation, bind to RNA-associated proteins or influence gene expression by regulating gene splicing or mRNA levels.^18^ However, whether gut microbiota alters tumor microenvironment by regulating non-coding RNAs, contributing to cancer metastasis, has been unexplored.

In this study, we found that ABX increased cancer metastasis. Furthermore, analysis of gut microbiota by deep sequencing combined with animal models and fecal transplantation identified a critical role of gut microbiota in the regulation of cancer progression and metastasis through IL-11/circRNA/miRNA/SOX9 axis.

## Results

### Dysbiosis of gut microbiota is associated with enhanced cancer metastasis

**Figure 1.**
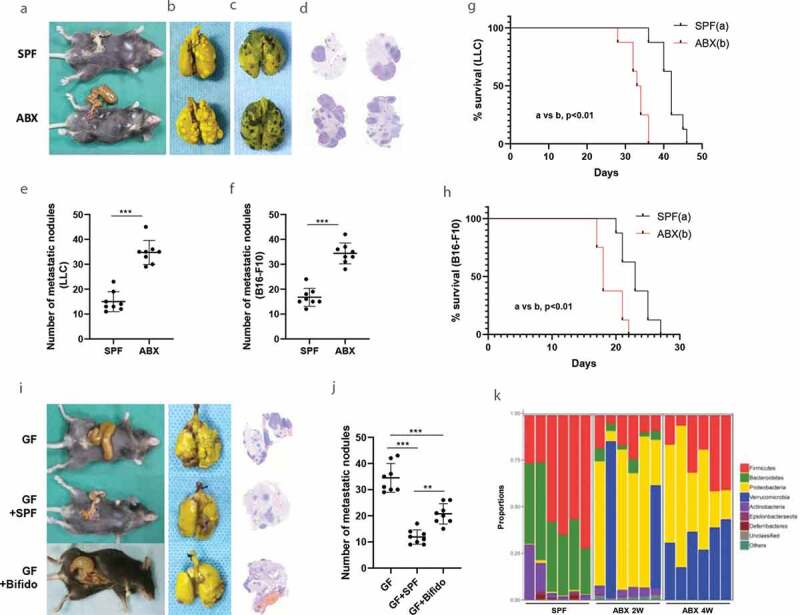
Gut microbiota dysbiosis promotes metastasis in animal experiment model. **A-F**, Tumor mice models were established by inoculating with LLC cells (b) or melanoma B16-F10 cells (c) via tail vein into C57BL/6 mice. The visible differences in the number of metastatic nodules between SPF and SPF/ABX mice were observed as a whole lung (b-c) or by pathological and HE stain (d). The number of metastatic nodules between different groups was calculated (e-f). **G-H**, Survival curves analysis. Kaplan-Meier estimates for survival rate of the SPF and SPF/ABX-treated mice. *p* values are shown [log-rank (Mantel-Cox) test analysis]. **I-J**, The visible differences in the number of metastatic nodules among GF, GF/SPF or GF/Bifido group were analyzed as a whole lung or by HE staining. These experiments were performed in two sets and 6–8 mice per group. **K**, By 16 S rDNA sequencing, mice fecal samples were sequenced to evaluate the influence of antibiotics on gut microbiota in different groups. Statistical analysis: one-way ANOVA (Figure 1E-F,1 J). Data are shown as mean ± SEM. *P < .05; **P < .01; ***P < .001.

To define the role of broad-spectrum antibiotics (ABX) mediated depletion of the gut microbiota in cancer metastasis, we used the syngeneic animal model. Microbial depletion, as supported by 16 S rDNA sequencing (Figure 1K, Fig. S1), was associated with cecum enlargement (Figure 1A). ABX application significantly promoted tumor metastasis. We also observed the visible differences in the number of metastatic nodules between SPF (mice raised in specific pathogen-free conditions) and SPF/ABX (SPF mice with broad-antibiotics administration) mice (Figure 1B-F). Survival analysis showed that the ABX application significantly reduced the survival rate (Figure 1G and H).

To determine the role of gut microbiota in tumor metastasis, we took two approaches. The first one was fecal microbiota transplantation (FMT) and the second one was Bifidobacterium inoculation because Bifidobacterium is known for its beneficial effect.^19^ We found that FMT from SPF mice or Bifidobacterium into germ-free (GF) avatar mice inhibited tumor metastasis. There were visible differences in the number of metastatic nodules between GF mice and GF/SPF (FMT using SPF mouse stool into the GF mice) group. These phenotypes were restored after FMT from SPF mice or Bifidobacterium into GF avatar mice (Figure 1I and J).

Next, we analyzed the composition of the gut microbiota of fecal specimens from ABX treated mice since ABX is known to influence gut microbiota.^20,21^ 16 S rDNA sequencing revealed that ABX significantly reduced the number and types of gut microbiota. For instance, compared with the no-ABX group, the Bifidobacterium was significantly reduced in the ABX group. By contrast, FMT using SPF mouse stool into the ABX mice for two weeks restored intestinal flora (Figure 1K, Fig. S1).

### Gut microbiota regulates the expression of miRNAs

Studies^22,23^ have suggested that non-coding RNAs, including miRNAs and circRNAs, play important roles in the regulation of tumor microenvironment. Given the possible involvement of gut microbiota in cancer metastasis, we asked whether gut microbiota can regulate the circulating miRNAs and even miRNAs in tumor tissue.

**Figure 2.**
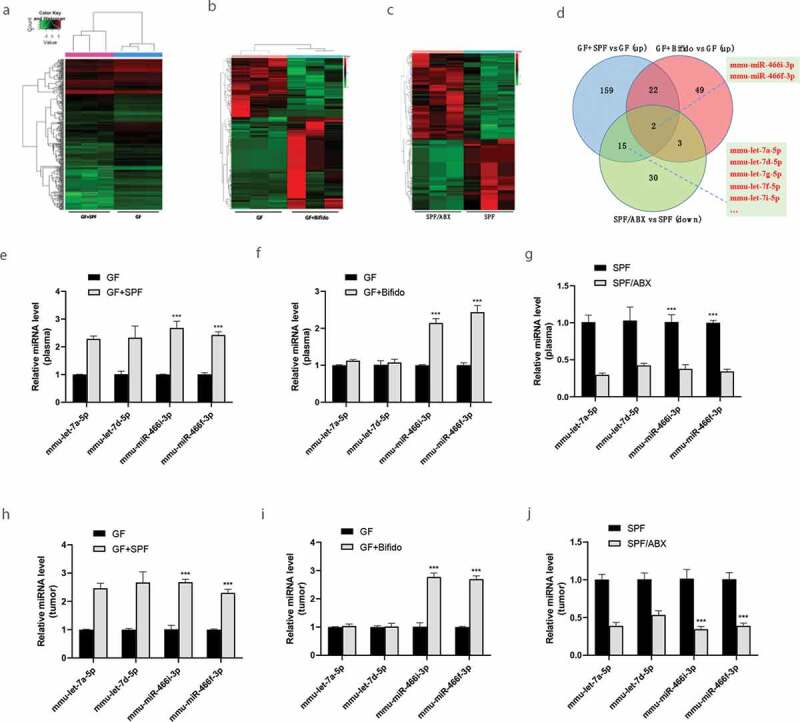
Gut microbiota regulates the expression of miRNAs. **A-C**, RNA deep sequencing analysis reveals differentially expressed circulating miRNAs between GF/SPF and GF (a), GF/Bifido and GF(b), SPF and SPF/ABX(c) mice. **D**, Target miRNAs including let-7 families and mmu-miR-466i-3p and mmu-miR-466 f-3p which are consistent in expression between GF/SPF and GF, GF/Bifido and GF, SPF and SPF/ABX groups. **E-G**, Validation of differentially expressed circulating miRNAs between GF/SPF and GF, GF/Bifido and GF, SPF and SPF/ABX mice by qRT-PCR. **H-J**, Validation of the expression levels of miRNAs in tumor tissue, which may regulate targeted mRNAs in tumor between GF/SPF and GF, GF/Bifido and GF, SPF and SPF/ABX mice by qRT-PCR. Statistical analysis: two-way ANOVA (Figure 2e-G, 2 H-J). Data are shown as mean ± SEM. *P < .05; **P < .01; ***P < .001.

We found important regulatory functions of circulating miRNA networks in a gut microbiota-dependent manner. For example, microarray and RNA sequencing identified differentially expressed miRNAs between GF and GF/SPF, GF and GF/Bifidobacterium, SPF and SPF/ABX mice. (Figure 2 A-C). miRNA microarray analysis for GF/SPF vs GF group detected a total of 1176 miRNAs, including 198 upregulated and 244 downregulated miRNAs. Similarly, miRNA sequencing analysis for GF/Bifido vs GF group detected a total of 898 miRNAs, including 116 upregulated and 75 downregulated miRNAs. For SPF/ABX vs SPF group also analyzed by miRNA sequencing, there were a total of 938 miRNAs, including 138 upregulated and 115 downregulated miRNAs.

Of particular interest, a subset of circulating miRNAs was induced by gut microbiota, and they were mmu-let-7a-5p, mmu-let-7d-5p, mmu-let-7 g-5p, mmu-let-7i-5p, mmu-let-7 f-5p, mmu-let-7e-5p, mmu-miR-466i-3p and mmu-miR-466 f-3p. Depletion of the microbiota with ABX in SPF mice significantly reduced these miRNAs (Figure 2C). Particularly, mmu-miR-466i-3p and mmu-miR-466 f-3p are shared between all GF/SPF and GF, GF/Bifido and GF, SPF and SPF/ABX groups (Figure 2D). Expression of several of these miRNAs in circulation (Figure 2E-G) was verified by qRT-PCR between GF and GF/SPF; GF and GF/Bifido; SPF and SPF/ABX mice. We further verified the expression levels of miRNAs in tumor tissue by qRT-PCR, supporting that these gut microbiota-dependent circulating miRNAs may be involved in the regulation of targeted mRNAs in tumor tissue (Figure 2H-J).

A subset of miRNAs was significantly regulated by FMT from SPF mice or intragastric administration with Bifidobacterium into GF mice. Moreover, we found that mmu-miR-466i-3p and mmu-miR-466 f-3p are shared between GF/SPF and GF, GF/Bifido and GF, SPF and SPF/ABX groups (Figure 2D), highlighting the importance of these gut microbiota-dependent miRNAs in maintaining the integrity of the internal environment.

### Gut microbiota regulates the expression of circRNAs

**Figure 3.**
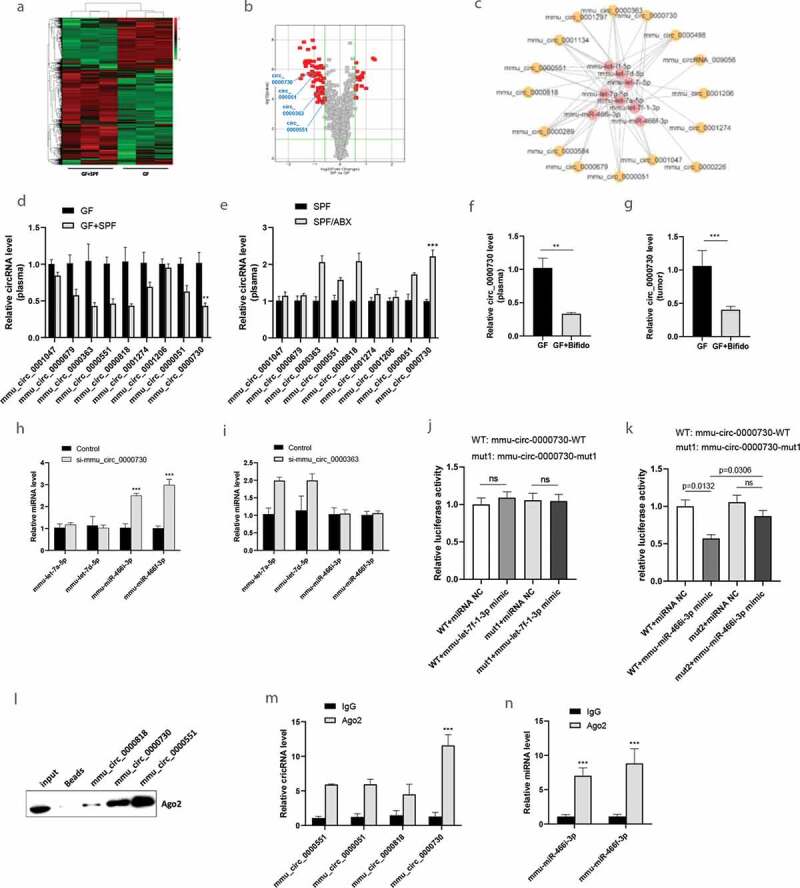
Gut microbiota regulates the expression of circRNAs. **A-B**, The circulating circRNA expression profile from GF/SPF and GF mice was compared using a high-throughput circRNA microarray. **C**, Interaction predictive analysis of target microRNAs and target circRNAs, which are regulated by gut microbiota. **D-E**, The differentially expressed circRNAs were identified, and qRT-PCR was used to verify a subset of the differentially expressed circulating circRNAs. **F-G**, Circulating mmu_circ_0000730 were detected in GF, GF/Bifido groups by RT-qPCR, and was further verified by qRT-PCR between GF and GF/Bifido group in tumor tissue. Statistical analysis: t test. **H-I**, Competitive regulation of mmu_circ_0000730 and mmu-miR-466i-3p/mmu-miR-466 f-3p. **J-K**, Dual-luciferase reporter assay:interaction analysis of mmu_circ_0000730 and mmu-miR-7 f-1-3p (j), mmu_circ_0000730 and mmu-miR-466i-3p (k). **L**, RNA pulldown by designed nucleic acid probes for target circRNAs to detect Ago2 protein in the RISC protein complex followed with western blot. **M-N**, RIP experiment with Ago2 antibody to check the target circRNAs including mmu_circ_0000730 (m) or miRNAs (n) by qRT-PCR. Statistical analysis: two-way ANOVA (D,E,H,I,J,K,M & N). Data are shown as mean ± SEM from three experiments performed in triplicates. *P < .05; **P < .01; ***P < .001; ns, not significant.

In addition to circulating miRNAs, we also found that circRNAs were differentially expressed in GF/SPF and GF mice (Figure 3A and B). For example, microarray analysis of GF/SPF vs GF group detected a total of 992 circRNAs, including 18 upregulated and 61 downregulated circRNAs. Interaction predictive analysis of target miRNAs (such as mmu-let-7a-5p, mmu-let-7d-5p, mmu-let-7 g-5p, mmu-let-7i-5p, mmu-let-7 f-5p, mmu-let-7e-5p, mmu-miR-466i-3p and mmu-miR-466 f-3p) identified that several circRNAs were potentially regulated by gut microbiota (Figure 3C) and verified by qRT-PCR (Figure 3D-E, Fig. S2A-2B). Several circRNAs were differentially expressed between GF and GF/SPF, SPF and SPF/ABX in plasma (Figure 3D and E) or tumor tissue (Fig. S2A and S2B). Mmu_circ_0000730 was significantly downregulated in the GF/SPF group compared to the GF group but significantly upregulated in SPF/ABX group compared to the SFP group. Compared to its expression in the GF group, circulating mmu_circ_0000730 was significantly downregulated in GF/Bifido group (figure 3F). Moreover, mmu_circ_0000730 was also significantly decreased in GF/Bifido tumor tissues compared to GF tumor tissues (Figure 3G).

Suppression of mmu_circ_0000730 by RNAi significantly increased mmu-miR-466i-3p and mmu-miR-466 f-3p expression, whereas suppression of mmu_circ_0000636 by RNAi significantly increased mmu-let-family (Figure 3H and I). Mmu_circ_0000730 is predicted to interact with mmu-miR-466i-3p and mmu-miR-466 f-3p, and sox9 is a direct target of mmu-miR-466i-3p and mmu-miR-466 f-3p. We found that mmu_circ_0000730 and mmu-miR-466i-3p or mmu-miR-466 f-3p had two complementary base sequences using bioinformatics analysis software RNA 22v2 (https://cm.jefferson.edu/rna22/). Luciferase reporters were constructed by inserting either the wild-type (WT) mmu_circ_0000730 sequence or the sequence with mutated (MUT) binding sites of mmu-miR-466i-3p (Fig. S3). We found that overexpression of mmu-miR-466i-3p decreased the luciferase activities of the wild-type reporter for mmu_circ_0000730, but not the activities of the mutant reporter (Figure 3J and K).

Moreover, Ago2 protein was captured in the biotin-labeled mmu_circ_0000730 group as compared to the control group (Figure 3l), suggesting that mmu_circ_0000730 could bind to mmu-miR-466i-3p in the RISC complex.^24^ In addition, compared with the control group, specific enrichment of mmu_circ_0000730, mmu-miR-466i-3p and mmu-miR-466 f-3p was detected in the Ago2 pull-down (Figure 3M and N).

### Effect of gut microbiota on tumor gene expression

**Figure 4.**
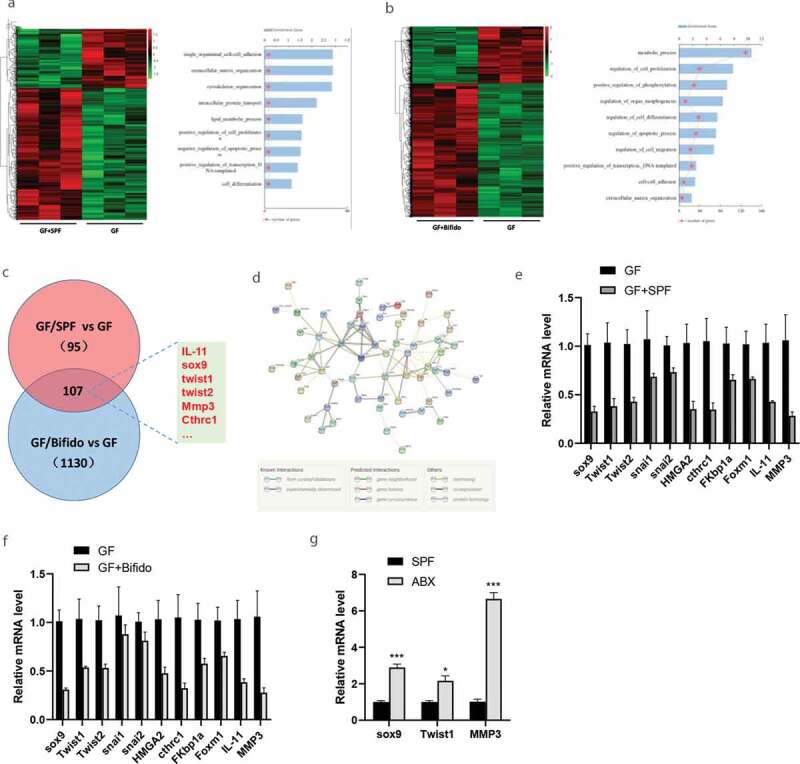
Gut microbiota regulates the expression of tumor genes. **A-B**, LLC Lewis cells were injected into C57BL/6 mice via tail vein to establish tumor model. The lung metastatic tumor nodules were isolated as samples from the mouse model for the molecular assays, and RNA deep Sequencing which revealed the alterations of gene expression between GF and GF/SPF, GF and GF/Bifido group. **C-D**, GO analysis and pathway analysis of the RNA deep sequencing result. Target genes including sox9, IL-11, twist and MMP3 are consistent in expression between GF and GF/SPF, GF and GF/Bifido groups. **E-F**, Validation of target gene expression between GF/SPF and GF, GF/Bifido and GF mice by qRT-PCR. **G**, Validation of target gene expression between SPF and SPF/ABX mice by RT-qPCR. Data are shown as mean ± SEM from three experiments performed in triplicate. *P < .05; **P < .01; ***P < .001. Statistical analysis: two-way ANOVA (E&F&G).

We then determined whether there is a correlation between the gut microbiota and cancer metastasis by colonizing 8-wk-old GF mice with the feces of age-matched SPF mice or Bifidobacterium into GF mice. Such colonization reversed the aggravated cancer metastasis in GF mice. We analyzed gene changes between GF and GF/SPF, GF and GF/Bifido mice after oral gavage of a combination of SPF feces colonization or Bifidobacterium in GF mice model, and found significant alterations of gene expression in those treatment groups (Figure 4A and B). RNA-seq analysis of the GF/SPF vs GF group detected a total of 11970 genes, and among them 28 genes were upregulated and 14 genes were downregulated. For RNA-seq analysis of the GF/Bifido vs GF group, there was a total of 12513 genes, including 828 upregulated and 234 downregulated genes.

Gene Ontology (GO) analysis suggested that the differentially expressed mRNAs might be involved in metabolic process, cell-cell adhesion, extracellular matrix organization, cell apoptosis, cell differentiation, cell proliferation, regulation of cell migration, and other biological functions (Figure 4A and B). Kyoto Encyclopedia of Genes and Genomes (KEGG) analysis suggested that the target genes of the differentially expressed mRNAs might be involved in the regulation of cancer stem cell, EMT, vascular endothelial growth factor, and other signaling pathways (Figure 4C and D). Compared with GF mice, FMT from SPF mice or intragastric administration with Bifidobacterium into GF mice (GF/SPF or GF/Bifido) downregulated expressions of genes, such as SRY-box transcription factor 9 (SOX9), interleukin (IL-11), twist1, and matrix metallopeptidase 3 (MMP3). These genes were altered in a gut microbiota-dependent manner (Figure 4E-F). Interaction analysis suggested that gut microbiota-mediated regulation of SOX9 was dependent on miRNAs (Fig. S12).

Colonization of GF mice with a normal microbiota inhibited cancer metastasis by suppressing the SOX9 pathway. For example, the levels of SOX9, Twist1 and MMP3 were significantly reduced by FMT from SPF mice or intragastric administration with Bifidobacterium into GF mice, but significantly induced by ABX treatment in SPF mice (Figure 4G). SOX9 has been shown to promote the stemness of cancer stem cell or cancer progression *in vitro* and *in* vivo.^25–28^

### Gut microbiota regulates cancer stem cells through circRNA/miRNA network

TargetScan analysis suggested that mmu_circ_0000730 can interact with mmu-miR-466i-3p and mmu-miR-466 f-3p. Furthermore, there were complementary sequences between mmu-miR-466i-3p and SOX9 which is a potential target for mmu-let-7a-5p, mmu-let-7d-5p, mmu-let-7 g-5p, mmu-let-7i-5p, mmu-let-7 f-5p, mmu-let-7e-5p, mmu-miR-466i-3p and mmu-miR-466 f-3p. As expected, suppression of mmu-miR-466i-3p and mmu-miR-466 f-3p increased SOX9 mRNA levels (Fig. S4A), whereas suppression of SOX9 induced these three miRNAs (Fig. S4B). Finally, suppression of mmu_circ_0000730 reduced the SOX9 mRNA level (Fig. S4 C).

**Figure 5.**
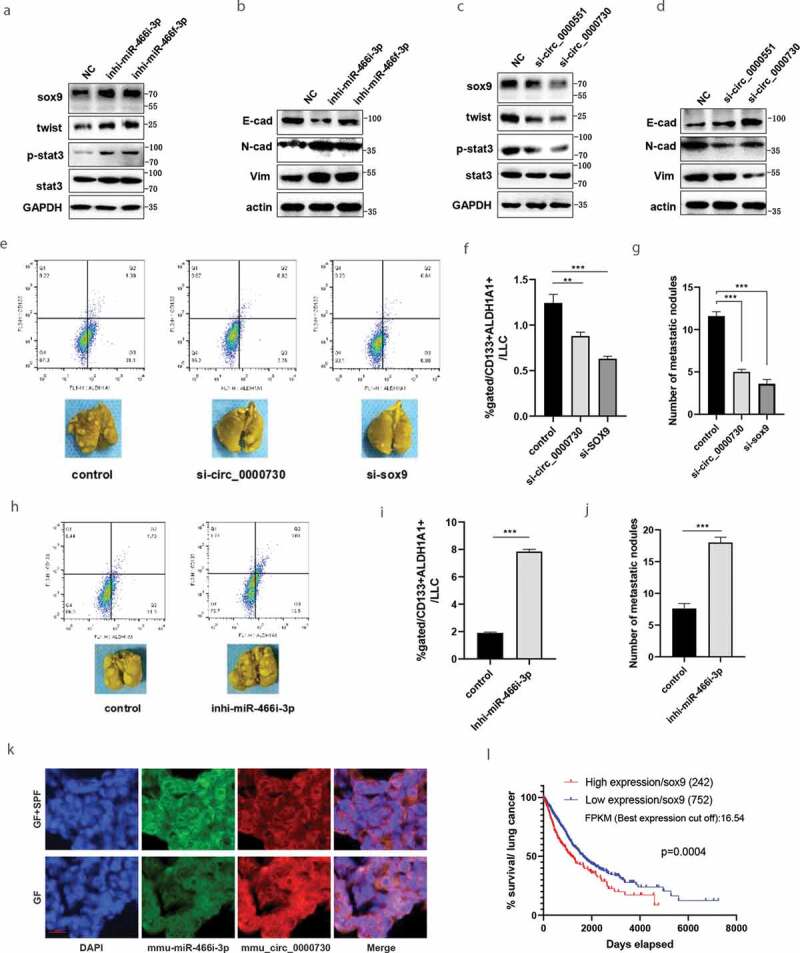
Gut microbiota regulates cancer stem cells through circRNA/microRNAs network. **A-D**, To determine whether miRNAs **(A & B)** or circRNAs **(C & D)** regulate the expression of target oncogenic genes such as SOX9 or epithelial-mesenchymal transition (EMT) marker genes such as N-cadherin, vimentin and E-cadherin. **E-G**, Regulatory role of sox9 or mmu_circ_0000730 on metastasis. Metastasis evaluations were performed in two sets and 6–8 mice per group, representative images (**E**, bottom) and cancer stem cell marker expression, CD133+ ALDH1A1+ cancer stem cells were detected by flow cytometry (**E**, upper, & **F**). **H-J**, Regulatory role of mmu-miR-466i-3p on metastasis. Metastasis evaluations were performed in two sets and 6–8 mice per group (H, bottom) and cancer stem cell marker expression, CD133+ ALDH1A1+ cancer stem cells were examined by flow cytometry (**H**, upper & **I**). **K**, FISH analysis of mmu_circ_0000730 or mmu-miR-466i-3p expression in LLC Lewis tumor tissues. Scale bar, 100 μm. **L**, Survival analysis by TCGA data (https://www.proteinatlas.org) showed that high expression of SOX9 with a significantly reduced survival rate in lung cancer patients. The thresholds of “high” and “low” were set according to FPKM (the best expression cut off). *P* values are shown [log-rank (Mantel-Cox) analysis]. Statistical analysis: one-way ANOVA (F & G), t test (I & J). Data are shown as mean ± SEM from three experiments performed in triplicate. *P < .05; **P < .01; ***P < .001.

Next, we determined whether miRNAs or circRNAs regulate the expression of SOX9 or epithelial-mesenchymal transition (EMT) marker genes. As shown in Figure 5A and B, the protein levels of SOX9, *p*-STAT3, Twist, N-cadherin and vimentin were increased by either mmu-miR-466i-3p or mmu-miR-466 f-3p inhibitors in LLC cells. However, the protein levels of E-cadherin were increased by either mmu-miR-466i-3p or mmu-miR-466 f-3p inhibitors in LLC cells. Moreover, the protein levels of SOX9, *p*-STAT3 Twist, N-cadherin and vimentin were reduced, and E-cadherin was increased by mmu_circ_0000730 siRNA in LLC Lewis cells (Figure 5C and D). These results suggest that mmu_circ_0000730 upregulates SOX9 expression and activates EMT markers by targeting mmu-miR-466i-3p or mmu-miR-466 f-3p.

To evaluate the effects of SOX9 or mmu_circ_0000730 on metastasis, CD133+ ALDH1A1+ cells were examined by flow cytometry. Down-regulation of SOX9 or mmu_circ_0000730 significantly reduced the proportion of CD133+ ALDH1A1+ cells (Figure 5E and F). To determine the role of mmu_circ_0000730 on metastasis, siRNAs of mmu_circ_0000730 or si-NC were transfected into LLC cells and the transfected cells were then injected into SPF mice by tail vein. siRNA of mmu_circ_0000730 or SOX9 caused a significant reduction of metastasis as compared to those in the control group (Figure 5E-G).

On the other hand, suppression of mmu-miR-466i-3p significantly increased the CD133+ ALDH1A1+ population (Figure 5H and I). Moreover, suppression of mmu-miR-466i-3p significantly promoted metastasis (Figure 5H-J) compared to control group. Fluorescence *in situ* hybridization (FISH) analysis showed that mmu_circ_0000730 and mmu-miR-466i-3p were co-localized in the LLC Lewis tumor tissues (Figure 5K). Moreover, down-regulation of SOX9 or mmu_circ_0000730 significantly reduced cancer cell invasion, while down-regulation of mmu-mi-466i-3p significantly increased cancer cell invasion (Fig. S5A~C). Survival analysis by TCGA data showed that high expression of SOX9 was significantly associated with the reduced survival rate in lung cancer patients (Figure 5l). Moreover, high expression of SOX9 is significantly associated with reduced survival rate in adenocarcinoma of the lung (LUAD) (Fig. S6A), squamous cell carcinoma of the lung (LUSC) (Fig. S6B), or melanoma (Fig. S6C) patients.

Altogether, these results suggest that mmu_circ_0000730 targets mmu-miR-466i-3p and promotes cancer progression by suppressing the oncogenic effects of SOX9, activating STAT3 and forming a mmu_circ_0000730/miRNAs/SOX9 axis.

### IL-11 plays a critical role in gut microbiota-mediated circRNA/miRNA/SOX9 axis

**Figure 6.**
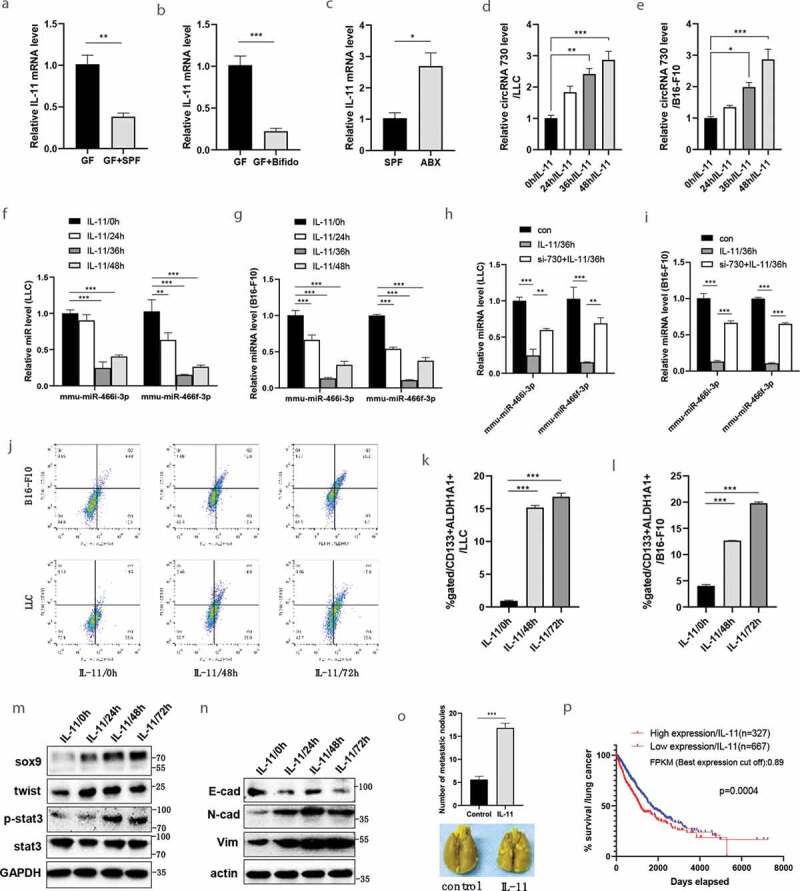
Gut microbiota regulates cancer metastasis through IL-11-mediated circRNA/microRNAs/sox9 axis. **A-C**, According to our RNA-seq results, transcript levels of IL-11 in tumor tissue were determined by qRT-PCR between GF and GF/SPF, GF and GF/Bifido, SPF and SPF/ABX groups. **D-E**, By LLC (d) or B16-F10 (e) cell line, to determine the effect of IL-11 on mmu_circ_0000730 expression by RT-qPCR. **F-G**, By LLC (f) or B16-F10 (g) cell line, to determine the effect of IL-11 on the expression of mmu-miR-466i-3p and mmu-miR-466 f-3p. **H-I**, IL-11 downregulates the expression of mmu-miR-466i-3p and mmu-miR-466 f-3p, while si-RNA-circ730 reverses these actions, by LLC (h) or B16-F10 (i) cell line. **J-L**, Regulatory role of IL-11 on cancer stem cells. CD133, ALDH1A1 alone or CD133/ALDH1A1 co-labeled cancer stem cells were examined by flow cytometry (**J, K&L**). **M**, IL-11 activates the STAT3 signaling pathway and promotes the expression of cancer stem cell marker genes such as SOX9 and twist in LLC cells. **N**, IL-11 promotes epithelial-mesenchymal transition (EMT) marker genes such as N-cadherin, vimentin and E-cadherin by western blot in LLC cells. **O**, IL-11 promotes tumor metastasis. Metastasis evaluations were performed in two sets and 6–8 mice per group, representative images (**O**, bottom). **P**, Survival analysis by TCGA data (https://www.proteinatlas.org) showed that high expression of IL-11 contributed to a reduced survival rate in lung cancer patients, *P* values are shown [log-rank (Mantel-Cox) analysis]. In Kaplan-Meyer plots by gene expression (IL-11), the thresholds of “high” and “low” were set according to FPKM (the best expression cut off). Data are shown as mean ± SEM from three experiments performed in triplicate (a-l). Statistical analysis: t test (A,B,C,O), one-way ANOVA (d,e), two-way ANOVA (F,G,H,I,K,L). *P < .05; **P < .01; ***P < .001.

RNA-seq showed that intestinal flora fecal microbiota transplantation (FMT) by intragastric administration of SPF fecal flora or by intragastric administration of Bifidobacterium into GF mice significantly affected the expression of IL-11 in tumors (Figure 4A-C). This result was further verified by qRT-PCR (Figure 4E-F). Compared with GF mice, intestinal flora reconstruction or Bifidobacterium administration in GF mice significantly decreased the IL-11 expression in tumor. In addition, compared with SPF mice, tumor IL-11 expression significantly increased in the SPF/ABX group (Figure 6A~ C). Similar results were also seen for circulating IL-11, as detected by qRT-PCR, between GF and GF/SPF, GF and GF/Bifido groups. (Fig. S7)

IL-11 treatment increased mmu_circ_0000730, while decreased mmu-miR-466i-3p and mmu-miR-466 f-3p expression (Figure 6D-G). Furthermore, IL-11 induced mmu_circ_0000730/SOX9 expression, while it downregulated the expression of mmu-miR-466i-3p and mmu-miR-466 f-3p: siRNA-circ730 reversed these actions (Figure 6H and I). In LLC cell line, there was no significant influence of IL-11 on the expression of Let-7 families (Fig. S8).

To determine whether IL-11 promotes tumor progression by enhancing the stemness of cancer stem cells, LLC Lewis or B16/F10 cells were treated with or without IL-11. FACS analysis revealed that IL-11 significantly increased the proportion of CD133+ ALDH1A1+ cells (Figure 6J-L), suggesting a role for IL-11 in the regulation of cancer stem cells. Furthermore, IL-11 induced cell invasion (Fig. S9). The qRT-PCR analysis showed that IL-11 promoted SOX9 expression in LLC (Fig. S10A) and B16-F10 (Fig. S10B) cell line. Finally, IL-11 promoted the stemness of cancer stem cell and EMT markers (Figure 6M and N), and intraperitoneal injection of IL-11 significantly promoted tumor metastasis (Figure 6O), which is consistent with the finding from the analysis of TCGA lung cancer dataset that high expression of IL-11 was associated with the poor survival rate of lung cancer patients (Figure 6P, Fig. S11).

## Discussion

Gut microbes may shape the response to anti-cancer therapy.^29,30^ Studies^5,7,31–36^ have highlighted the key role of the gut microbiota in mediating tumor responses to chemotherapeutic agents or immunotherapies targeting PD-L1 or cytotoxic T lymphocyte-associated protein 4 (CTLA-4). Thus, maintaining healthy gut flora could help patients combat cancer, and as such, it is possible to improve therapeutic response by modulating the microbiome.^37–39^

The integrity of gut microbiota or Probiotics Bifidobacterium is favorable in anti-cancer therapy,^40,41^ however, cancer patients often take ABX generally for common indications (such as pneumonia or urinary tract infection),^42,43^ or take ABX during the perioperative period, or because of diagnostic treatment in order to exclude infection before the definite diagnosis. Independent of classical prognostic markers in advanced non-small cell lung cancer (NSCLC), ABX uptake has a negative impact on overall survival (OS) during chemotherapy.^3^ In addition, platinum chemotherapy combined with antibiotics can reduce cancer regression and survival in mice, whereas cisplatin combined with Lactobacillus bacteria can improve treatment response.^44^ Therefore, ABX may represent a predictor of resistance to chemotherapy or immunotherapy. However, how gut microbiota affects this process is still unclear, and the role of gut microbiota dysbiosis in cancer metastasis and the underlying mechanisms remain largely unknown.

In the present study, using mouse tumor models established by LLC or B16-F10 cells, we show higher aggravated metastasis and lower survival rate in ABX mice or germ-free mice than in SPF mice. By contrast, fecal microbiota transplantation (FMT) by intragastric administration of SPF fecal flora or Probiotics Bifidobacterium significantly alleviates lung metastasis. Therefore, these results suggest that gut microbiota plays crucial roles in the regulation of cancer metastasis,

Analyzes of gut microbiota in experimental animals suggest that ABX significantly influences gut microbiota. Further studies suggest that gut microbiota impacts crucial regulatory functions of circRNA/miRNA networks that may contribute to cancer metastasis. For example, mmu_circ_0000730 is significantly upregulated in ABX treated mice or GF mice. Functionally, mmu_circ_0000730 siRNA inhibits the invasion and migration of tumor cells. Moreover, RNA precipitation and FISH as well as the dual-luciferase reporter assays suggest that mmu_circ_0000730 interacts with mmu-miR-466i-3p or mmu-miR-466 f-3p. Functional experiments and Western blotting reveal a correlation between mmu_circ_0000730, mmu-miR-466i-3p and SOX9. These results support the notion that mmu_circ_0000730 acts as a sponge for mmu-miR-466i-3p and mmu-miR-466 f-3p, which targets SOX9. In this way, mmu_circ_0000730 maintains a high level of SOX9 and promotes the stemness of cancer stem cell and EMT by titrating the function of mmu-miR-466i-3p and mmu-miR-466 f-3p.

In this study, we have used both microarray and next-generation sequencing (NGS) for gene profiling. While microarray technology was developed early days, NGS is a relatively new technology. Based on a comparative study,^45^ there is a high correlation between gene expression profiles generated by these two platforms. The advantages of microarray technology are high throughput, relatively quick, and sensitive at low cost. However, limitations for microarrays include a need for predesigned probes on the chip and thus it can only detect known genes. Other limitations associated with microarrays are cross-hybridization, nonspecific hybridization, and limited detection range of individual probes. On the other hand, NGS does not need predesigned probes, which would be able to identify new genes. In particular, NGS is better in detecting low abundance transcripts, differentiating biologically critical isoforms, and allowing the identification of genetic variants. Finally, NGS is capable of detecting a broader dynamic range than microarrays. Because of these features, NGS has become a predominant platform. That is why we adopted NGS for the experiments at the later stage of our study.

CircRNAs are endogenous RNAs that have gene regulatory functions;^46,47^ they are characterized by its stable expression, long half-life, and specific expression in different tumors, and as a novel tumor biomarker for tumors.^48^ In contrast to linear RNA, circRNAs are formed by covalently closed-loop structures with unique structures and high stability or diversity.^49^ In recent years, circRNAs have been shown to play a role in many biological processes and the progression in many diseases.^50,51^ The major function of circRNAs seems to negatively regulate miRNA activity, resulting in the regulation of miRNAs which subsequently regulate the expression of target genes.^52^

Increasing evidence^53^ has suggested that non-coding RNAs carried in exosomes can travel in a long distance in the circulating system and can reach various tumor sites. It is well known that microbiome do not directly interact with tumor cells. Thus, a question is how microbiome would regulate tumor miRNA expression. Our profiling data on the circulating non-coding RNAs regulated by microbiome suggests that microbiome may have impact first on circulating non-coding RNAs and then on tumor tissue through exosome-mediated gene transfer, thus providing at least a possible link between microbiome and tumors.

Our study further reveals a crucial role for IL-11 in this regulatory system. For example, colonization of fecal microbiota transplantation (FMT) from SPF mice or intragastric administration of Bifidobacterium into GF mice or ABX mice reduces cancer progression, along with the release of IL-11. IL-11 is a member of the IL-6 family, and they share gp130 as the common signal transducer.^54^ Studies have shown that IL-11 plays important roles in cancer regulation. For instance, IL-11 activates STAT3 in cancer-associated fibroblasts, and promotes colorectal tumor development, and correlates with poor prognosis.^55^ In breast cancer, tumor cell-derived IL-11 may promote osteolysis by increasing the pool of osteoclast progenitor cells.^56^ IL-11 is also essential in promoting osteolysis in breast cancer bone metastasis.^57^ Thus, it is likely that IL-11 release may serve as the first step after dysbiosis of the gut microbiome, followed by activation of the SOX9 in ABX or GF mice. Therefore, there is a mechanistic link between the gut microbiota and cancer metastasis through the IL-11/circRNA/miRNA axis.

Together, these results may have a significant implication for understanding a novel gut microbiota-mediated regulatory system in cancer metastasis. This demonstrates a strong interaction between the microbiota and cancer, which indicates potential mechanisms linking microbial dysbiosis to cancer progression.^58,59^ Moreover, cancer patients often face stresses that can cause gut barrier dysfunction and systemic endotoxemia. By reinforcing intestinal barrier integrity and reducing systemic inflammation, the patient might generate “homeostatic” consortia of commensals that prevent leaky colon and systemic immunosuppression.^60^ For example, oral feeding with Bifidobacterium alone in our study significantly affects circRNA/microRNA networks and cancer metastasis in GF mice, suggesting that gut microbiota play a regulatory role by more complex mechanisms. Therefore, gut microbiota could modulate cancer metastasis in a circRNA/miRNA dependent fashion, which will help to pave the way to clinical translation of the use of gut microbiota for cancer prevention or treatment in the near future.^61,62^

In summary, our study identifies a critical role of gut microbiota in the regulation of cancer metastasis. Our study further suggests that gut microbiota-dependent circRNAs form a large class of post-transcriptional regulatory networks with miRNAs, a previously unrecognized regulatory role of non-coding RNAs in cancer metastasis in an endogenous microbiota-dependent manner. Therefore, a better understanding of this regulatory system will help develop strategies for improving chemotherapy efficacy or circumventing primary resistance to chemotherapy/immunotherapy by manipulation of the gut ecosystem.

## Materials and methods

### Reagents

Primary antibody against SOX9 (ab185230), Twist (ab49254), STAT3 (ab119352), *p*-STAT3 (ab30647), E-cad(ab181296), N-cad(ab76011), Vimentin (ab137321), ALDH1A1(ab52492), Ago2 (ab32381),GAPDH (ab8245) and actin(ab5694) were purchased from Abcam; MMP3 (Catalog,17873-1-AP) from Proteintech(Chicago, IL); CD133 (PE, eBioscience) from Invitrogen; IL-11 (Catalog Number. 220–11) from Peprotech. Secondary antibodies were purchased from Invitrogen. Pooled siRNAs against SOX9, mmu_circ_0000730, mmu_circ_0000363, mmu_circ_0000051, mmu_circ_0000551, and control siRNA were purchased from RiboBio (Guangzhou, China). Pooled biotin-labeled probes for RNA-pulldown against mmu_circ_0000730, mmu_circ_0000818, mmu_circ_0000551 and control oligos were purchased from RiboBio (Guangzhou, China). Inhibitors against mmu-let-7a-5p, mmu-let-7d-5p, mmu-let-7 g-5p, mmu-let-7i-5p, mmu-let-7 f-5p, mmu-let-7e-5p, mmu-miR-466i-3p and mmu-miR-466 f-3p, and control inhibitor were purchased from RiboBio (Guangzhou, China). PCR primers were purchased from RiboBio (Guangzhou, China). Probes for FISH against mmu_circ_0000730, mmu-miR-466i-3p were purchased from RiboBio (Guangzhou, China).

### Mice

All animal procedures with all protocols receiving ethical evaluation and were approved by the Institutional Animal Care and Use Committee (IACUC) of Tongji Hospital of Tongji University. All studies were performed in accordance with the NIH Guide for the Care and Use of Laboratory Animals. Male C57BL/6 mice purchased from Shanghai SLAC Laboratory Animal Co., Ltd (China) were bred and maintained in a specific pathogen-free environment, and generally used between 6 and 16 weeks of age. Germ-free C57BL/6 mice were bred at Shanghai Laboratory Animal Center of Chinese Academy of Sciences and maintained in the gnotobiotic facility.

For ABX mice models, mice were kept in specific pathogen-free (SPF) conditions and divided into two groups: a. SPF group; b. ABX group (Three weeks after drinking water with broad-spectrum antibiotics (ABX), followed with tumor cells being injected into C57BL/6 mice via tail vein, and ABX drinking water was continued until the mice were sacrificed.^3^

For IL-11 administration,^63^ 6–8 mice per group were i.p. injected daily with either recombinant mouse IL-11 (0.5 μg/mouse) (Peprotech),^63^ or control vehicle (PBS). The mice were monitored until the mice were sacrificed, followed with metastasis evaluation and histological studies. After tumor inoculation in mice, animals were monitored and harvested at day 21 (B16-F10) or day 28 (LLC) after injection, and lung metastasis evaluation was performed. The lung metastatic tumor nodules were isolated for the following molecular assays.

### Cell culture

The Lewis lung carcinoma (LLC) and melanoma B16-F10 cells were provided by the cell bank of Chinese Academy of Sciences (Shanghai). Both cell lines were tested for mycoplasma and the results were all negative (Fig. S18). B16-F10 cells were cultured in phenol-free RPMI 1640 (Hyclone) supplemented with 10% fetal bovine serum (Sigma-Aldrich) and 1% penicillin and streptomycin. LLC cells were grown in DMEM (Hyclone) supplemented with 10% fetal bovine serum (Sigma-Aldrich) and 1% penicillin and streptomycin. Cells were incubated at 37°C and supplemented with 5% CO2 in the humidified chamber. For the cell experiments including FACS analysis, LLC or melanoma B16-F10 cells were cultured in the absence or presence of IL-11 (Peprotech) (100 ng/ml) at different time points.^63^

### Predicting the target circRNAs and miRNAs of SOX9

To predict the target circRNAs of SOX9 using bioinformatics analysis, we adopted different data analysis tools, including circBase (http://www.circbase.org/), CircNet, and CircInteractome (https://circinteractome.nia. nih.gov/). Subsequently, we selected nine potential circRNAs. qRT-PCR confirmed that mmu_circ_0000730 exhibited significantly different expression (*p* < .01). We focused on mmu_circ_0000730 in this study. To predict potential miRNAs for SOX9, we used TargetScan and found a possible association between let-7 families or mmu-miR-466i-3p and mmu-miR-466 f-3p families and SOX9. We then used RNA 22v2 software to detect the binding sites between mmu-miR-466i-3p/mmu-miR-466 f-3p and mmu_circ_0000730. Finally, we determined mmu-miR-466i-3p/mmu-miR-466 f-3p with the highest loop score as the final miRNA.

### Construction of plasmids

PCR reactions for cloning purposes used high fidelity enzyme Phusion (Thermo Fisher Scientific). For luciferase assays, respective fragments of mmu_circ_0000730-wt (wild type) or mmu_circ_0000730-mut (mutant) were separately cloned into PHY-811 vector (RiboBio, Guangzhou, China) at Xho I and Not I sites. All PCR products were verified by DNA sequencing.

### Transfection

Cells were transfected with siRNAs or inhibitors and control oligos (siRNA-NC or inhibitor-NC) using RNAifectin reagent (Applied Biological Materials, Richmond, BC, Canada) or plasmid DNA using following the manufacturer’s protocol.

### RNA isolation, RT-PCR and RT-qPCR

We isolated total RNA including circulating IL-11 RNA using RNAiso Plus/Blood (Takara) per the manufacturer’s protocol and used 0.5 μg RNA to synthesize cDNA by PrimeScript RT reagent Kit (Perfect Real Time) (Takara). The concentrations of the RNA samples were determined by OD260 using a NanoDrop ND-1000 instrument. The resultant cDNA was used for PCR reactions using the following the manufacturer’s protocol. PCR annealing temperature varied depending on the primers used. To specifically detect the expression of target coding and circRNA genes, we used the SYBR Green method with primers described previously.^64^ GADPH was used as an internal control. Moreover, qRT-PCR for miRNAs was performed using miDETECT A Track miRNA qRT-PCR Starter Kit (Ribobio, China) following the manufacturer’s protocol. Delta-delta Ct values were used to determine their relative expression as fold changes, as previously described.^65^ The qRT-PCR results of expression levels of target genes shown as relative values.

### Western blot

Cells were harvested and protein was extracted from cells as previously described.^24^ The protein concentration was determined using BCA Protein Assay Kit (Beyotime Biotechnology) and samples were separated in sodium dodecyl sulfate-polyacrylamide gels. Original scans of the blots in the Source Data file as supplementary figures.

### RNA precipitation

To determine Ago2 protein in the RISC complex associated with target circRNAs and miRNAs, we performed RNA precipitation assay using synthesized target circRNAs including mmu_circ_0000730 as probes. Synthesized target circRNAs probes listed in Supplementary Table 4. The procedure was performed using the Bes5102 RNA pulldown kit (Bersinbio, Guangzhou) according to the manufacturer’s protocol. The labeled RNA was purified by a column-based kit (Zymo Research). The cellular extract was prepared from a 10 cm dish culture (~80% confluence) with a cell lysis buffer. For precipitation assays, the reaction (RNA probe and cellular extract) was incubated at 4°C for 60 min, followed by 5 washes with PBS. The pellets were used either for extraction of RNA for qRT-PCR or Western according to standard procedures.

### RNA immunoprecipitation (RIP)

To determine the interaction of target circRNAs or miRNAs in the Ago2 involved RNA-induced silencing complex (RISC), we used the Ago2 antibody for pulldown assays and then detected target circRNAs or miRNAs. Magna RIP™ RNA-Binding Protein Immunoprecipitation Kit (Millipore) was used for RIP procedures according to the manufacturer’s protocol. After the antibody was recovered by protein A + G beads, standard qRT-PCR was performed to detect RNA levels in the precipitates.

### 16 S rDNA sequencing

DNA extraction and PCR amplification: Microbial DNA was extracted from mouse feces specimen using the E.Z.N.A.® Soil DNA Kit (Omega Bio-Tek, Norcross, GA, U.S.) according to the manufacturer’s protocols. The V4-V5 region of the bacteria 16 S ribosomal RNA gene was amplified by PCR (95°C for 2 min, followed by 25 cycles at 95°C for 30 s, 55°C for 30 s, and 72°C for 30 s and a final extension at 72°C for 5 min) using primers 515 F 5ʹ-barcode- GTGCCAGCMGCCGCGG)-3ʹ and 907 R 5ʹ-CCGTCAATTCMTTTRAGTTT-3ʹ, where the barcode is an eight-base sequence unique to each sample. PCR reactions were performed in triplicate 20 μL mixture containing 4 μL of 5 × FastPfu Buffer, 2 μL of 2.5 mM dNTPs, 0.8 μL of each primer (5 μM), 0.4 μL of FastPfu Polymerase, and 10 ng of template DNA. Amplicons were extracted from 2% agarose gels and purified using the AxyPrep DNA Gel Extraction Kit (Axygen Biosciences, Union City, CA, U.S.) according to the manufacturer’s instructions and quantified using QuantiFluor™ -ST (Promega, U.S.).

Library construction and sequencing: purified PCR products were quantified by Qubit®3.0 (Life Invitrogen) and every twenty-four amplicons whose barcodes were different were mixed equally. The pooled DNA product was used to construct Illumina Pair-End library following Illumina’s genomic DNA library preparation procedure. Then the amplicon library was paired-end sequenced (2 × 250) on an Illumina HiSeq platform (Shanghai BIOZERON Co., Ltd) according to the standard protocols.

### Microarrays and RNA-seq

Total RNA was isolated using TRIzol (Invitrogen). The concentrations of the RNA samples were determined by OD260 using a NanoDrop ND-1000 instrument. The integrity of RNA was assessed by electrophoresis on a denaturing agarose gel. Total RNA quantification and quality assurance by spectrophotometer. For the spectrophotometer, the O.D. A260/A280 ratio should be close to 2.0 for pure RNA (ratios between 1.8 and 2.1 are acceptable). The O.D. A260/A230 ratio should be more than 1.8. RNA Integrity test by denaturing agarose gel electrophoresis. The 28 S and 18 S ribosomal RNA bands should be fairly sharp, intense bands. The intensity of the upper band should be about twice that of the lower band. RNA-seq and microarrays analysis was conducted by Aksomics (China).

The mRNA was enriched using NEB Next® Poly(A) mRNA Magnetic Isolation Module to total RNA. Then RNA-seq library was prepared using RNA KAPA Stranded RNA-Seq Library Prep Kit (Illumina). Libraries were quantified on the Agilent 2100 bioanalyzer and sequenced on the Illumina Hiseq 4000. Sequenced reads were 150bp long with paired-ends. The quality of the raw sequence data was assessed using FastQC and then the sequenced paired-end reads were aligned to the mouse reference genome (GRCm38). The FPKM matrix was log2 transformed and normalized among the groups. R software package was used for further data analyses.

For microRNA, the miRCURY™ Hy3™/Hy5™ Power labeling kit (Exiqon, Vedbaek, Denmark) was used to label miRNA. The Hy3™-labeled samples were hybridized on the miRCURYTM LNA Array (v.19.0) (Exiqon) according to the array manual. Then the slides were scanned using the Axon GenePix 4000B microarray scanner (Axon Instruments, Foster City, CA). GenePix Pro 6.0 software (Axon) was used to extract data and R software package was used for further analyses.

We performed microarray for miRNAs detection (GF vs. GF/SPF), RNA-seq for miRNAs (GF vs. GF/Bifido, SPF vs. SPF/ABX) and mRNAs (GF vs. GF/SPF, GF vs. GF/Bifido) detection.

Circular RNA was enriched with the use of RNase R to total RNA. The enriched circular RNA was then amplified and transcribed into fluorescent cRNA utilizing random primer according to Arraystar Super RNA Labeling protocol (Arraystar, Inc.). Then the labeled circRNAs were hybridized onto the Arraystar Mouse circRNA Arrays (8x15 K, Arraystar), and incubated for 17 hours at 65°C in an Agilent Hybridization Oven. Slides were scanned with the Agilent Scanner G2505 C after washing. Agilent Feature Extraction software was used to extract data. Quantile normalization of the data was then performed using the R software package. The circRNAs that at least 1 out of 6 samples have flags in “P” or “M” (defined by GeneSpring software) were retained for further differential analyses.

Relevant microarray and RNA-seq data have been uploaded: The miRNA microarray data have been deposited to the NCBI GEO database, accession GSE140339. The circRNA microarray data have been deposited to the NCBI GEO database, accession GSE140338. The RNA-seq data of mRNA (accession GSE140885) and miRNA (accession GSE140886) have been deposited to the NCBI GEO database. The 16 S sequencing data have been deposited to the NCBI Sequence Read Archive (SRA) database (Accession Number: SRP226777).

### Luciferase assays

Luciferase assays were performed using Dual-Luciferase Reporter Assay System (E1910) (Promega, Madison, WI) according to the manufacturer’s protocol. Briefly, Lewis lung carcinoma (LLC) cells were first transfected with appropriate plasmids in 12-well plates, and then cultured. Three days after transfection, the cells were harvested and lysed for luciferase assays. Renilla luciferase was used for normalization.

### Flow cytometry

LLC and B16-F10 melanoma cells were cultured in DMEM (Hyclone) or RPMI 1640 (Hyclone) supplemented with charcoal-stripped 10% FBS in duplicate in a 12-well plate at 37°C and supplemented with 5% CO2 in the humidified chamber. Experiments were performed in triplicates: LLC cells were transfected with siRNA of mmu_circ_0000730 or inhibitor of mmu-miR-466i-3p along with the negative control, while LLC or melanoma B16-F10 cells were stimulated with 100 ng/ml IL-11 (Peprotech) for 48 h or 72 h. Then cells were fixed and permeabilized with BD Cytofix/Cytoperm kit and stained with the corresponding antibodies. CD133 (PE) and ALDH1A1 (FITC) staining were performed according to the manufacturer’s instruction. Isotype controls were used to determine the background. The percentage of cells expressing each molecule was determined in gated cells, and data were analyzed by BD FACS Calibur Flow Cytometer (Becton-Dickinson, USA).

### Histology analysis

Histological examination of tumors for quantification was performed on 10% neutralized buffered formalin-fixed paraffin-embedded sections of lung metastatic tumors stained routinely with hematoxylin and eosin. Nodules were calculated and analyzed individually by a pathologist.

### Fluorescence in situ hybridization (FISH)

FISH was used to detect target circRNAs or miRNAs levels in LLC cells. Biotin-labeled antisense LNA probes derived from mmu_circ_0000730 and mmu-miR-466i-3p were listed in Supplementary Table 4. The procedure was performed using FISH Tag™ RNA Multicolor Kit (Invitrogen) according to the manufacturer’s protocol.

### Invasion assays

In order to measure the invasion ability of the cancer cells, transwell chambers (Corning, Inc., USA) with a polycarbonate filter and an 8 μm pore size, which were pre-coated by the matrix (BD, Biocoat) per the manufacturer’s protocol, were used in this study. In brief, medium containing 10% FBS (Sigma-Aldrich) was added to the bottom chamber as a chemoattractant. Transfected cells (5 × 10^4^ in 200 μl of serum-free medium) were seeded in the upper chamber and incubated at 37°C and supplemented with 5% CO2 in the humidified chamber. After 12–16 h, cells in the upper chamber were carefully removed by a cotton swab, and the cells on the opposite side of the filter were fixed with 70% ethanol for 30 min following stained with 0.1% crystal violet for about 10 min, and the migrated cells were calculated under a microscope (Leica, Germany).

### Administration of antibiotics

The c57bl/6 male mice were administered a cocktail of broad-spectrum antibiotics (ABX) in their drinking water. The cocktail consisted of vancomycin (500 mg/L), imipenem/cilastatin (500 mg/L) and neomycin (1 g/L) in drinking water as an antibiotic cocktail, and fresh antibiotics were administered every 3 days^3^. Fresh feces were collected at the beginning of the experiment, and then at different time points. DNA was extracted from the feces using Macherey-Nagel Nucleospin® Soil kit, following the manufacturer’s instructions. 500 pg of DNA was used to perform qPCR of the 16 S rDNA and quantify the number of bacterial copies, using E. coli purified 16 S rDNA as a standard.^66^

### Colonization with bacterial inocula

Mice were inoculated by gavage with SPF mouse stool or probiotics. Cecum and colon contents from age-matched c57bl/6 male mice were dissolved in 5 ml sterile, O2-free reduced PBS. Fecal microbiota transplantation (FMT) using SPF mouse stool was performed according to De Vadder F,^66^ animals were administered a single oral gavage of 200 µl of the cecum content solution. In a different set of experiments, the probiotics were resuspended in anaerobic PBS. C57BL/6 male mice were inoculated i. g. with 200 μl of probiotics (dose range: 2 × 10^8^ to 5 × 10^9^ CFU), 3 times per week, 2 weeks before the tumor injection, and during the trials. Colonization of feces was monitored, and feces specimens were collected during and at the end of the study.

### Mouse model

All procedures in mice were approved by the Ethics Committee on Animal Care and Use of Tongji hospital of Tongji University. Male c57bl/6 mice at 6–8 week old purchased from Shanghai SLAC Laboratory Animal Co., Ltd (China) were housed in an SPF condition or germ-free condition, with free access to water and food. LLC or B16-F10 cells were injected into these mice with 1 million B16-F10 or LLC cells (including control siRNA or target siRNAs) in 100 μl sterile PBS via tail vein, and 6–8 animals per group. Animals were monitored and harvested on day 21 (LLC) or day 28 (B16-F10) after injection. The two-group t-test was used to compare two means at each time point. All animals were included for analysis. In a different set of experiments, mice were randomly grouped. 8 mice per group were i.p. injected daily with either recombinant mouse IL-11 (0.5 μg/mouse) (Peprotech)^63^ or control vehicle (PBS), 3 times per week. The mice were monitored, until the mice were sacrificed, followed by histological studies.

### Statistical Analysis

All experiments from cell lines were performed using at least three independent experiments in triplicate. Means were calculated from at least three independent experiments. All results were shown as the means ± standard errors of the means (SEM). Two-sample t-test, Log-rank test, or Chi-squared test were used for statistical analyses. Data were analyzed by GraphPad Prism software (Version 8.0, GraphPad Prism Software Inc., San Diego, CA), and a *P*-value <0.05 was considered significant.
